# Comparison of Two Different Percutaneous Screw Fixations for Treating Herbert B2‐Type Acute Scaphoid Fractures

**DOI:** 10.1111/os.13517

**Published:** 2022-10-17

**Authors:** Haoliang Hu, Xiaofeng Teng, Xueyuan Li, Miaozhong Li, Shimin Chang

**Affiliations:** ^1^ Department of Orthopaedic Surgery Yangpu Hospital, School of Medicine, Tongji University Shanghai China; ^2^ Department of Hand Surgery Ningbo No. 6 Hospital Ningbo China

**Keywords:** bone, carpal arthroscopy, percutaneous screw internal fixation, scaphoid fracture, scaphoid nonunion

## Abstract

**Objective:**

Scaphoid fracture was the most common carpal fracture and the most challenging. The purpose of this study was to investigate and compare the clinical effects of closed reduction and percutaneous cannulated screw internal fixation under fluoroscopy and arthroscopy‐assisted percutaneous cannulated screw internal fixation in the treatment of Herbert B2‐type of acute scaphoid fractures.

**Methods:**

A retrospective controlled study was conducted on 29 patients with Herbert B2‐type acute scaphoid fracture with a displacement of >1 mm admitted to our hospital from January 2017 to June 2021. Patients were divided into two groups, 11 patients were treated with closed reduction percutaneous cannulated screw internal fixation under fluoroscopy and 18 patients were treated with percutaneous cannulated screw internal fixation assisted by arthroscopy. The operative time, intraoperative fluoroscopy times, fracture healing time, complications, and postoperative wrist function score of the two groups were compared.

**Results:**

All patients were followed up for 6–18 months (mean follow‐up duration: 10.38 ± 2.69 months). The respective operation times in the arthroscopy group and fluoroscopy group was 51.50 ± 6.69 min and 56.73 ± 11.48 min, respectively (*p* > 0.05). The number of fluoroscopies performed in the arthroscopy group was (6.83 ± 1.30), which was less than that in the fluoroscopy group (10.91 ± 2.62) (*p* < 0.05). All fractures in the arthroscopy group healed after the operation, and the fracture healing time was 11.44 ± 1.25W. Ten patients in the fluoroscopy group healed. The fracture healing time was 13.60 ± 2.32 W. The fracture healing time in arthroscopy group was less than that in the fluoroscopy group (*p* < 0.05). One patient in the fluoroscopy group had nonunion and healed after bone grafting and internal fixation. At the postoperative 6‐month follow‐up, the modified Mayo wrist function score was used to evaluate the clinical results. The wrist function score of patients in the arthroscopy group was 90 (85, 95), which was >80 (80, 90) in the fluoroscopy group (z = 2.74, *p* < 0.05).

**Conclusion:**

For Herbert B2‐type acute scaphoid fracture with fracture displacement > 1 mm, the arthroscopy‐assisted percutaneous cannulated screw internal fixation has less fluoroscopy times, short fracture healing time, and good recovery effect of wrist function compared to the fluoroscopy.

## Graphical abstract

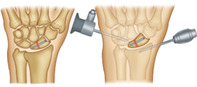

For Herbert B2‐type acute scaphoid fracture with fracture displacement >1 mm, compare to closed reduction percutaneous cannulated screw internal fixation, arthroscopic‐assisted percutaneous cannulated screw internal fixation has less fluoroscopy times, short fracture healing time, good recovery effect of wrist function, and less complications.

## Introduction

Scaphoid fracture is the most common carpal fracture,^1^, ^2^ accounting for 60%–70% of carpal fractures and 2% of total body fractures, which is only second to distal radius fracture.^3^ The blood supply system of scaphoid fracture is unique, and improper treatment in the early stage may lead to delayed healing or non‐healing of fracture, thus affecting wrist function.^4^ Traditional plaster fixation can be used for nondisplaced scaphoid fractures, but nonunion occurs in 15% cases.^5^ As for displaced fractures, the incidence of nonunion with plaster fixation could be as high as 50%.^6^, ^7^ Meta‐analysis suggests that the risk of nonunion caused by non‐surgical treatment is four‐times higher than that of non‐displaced fractures.^8^ Therefore, in recent years, surgical treatment is advised for displaced scaphoid fractures. However, open reduction and internal fixation can aggravate structural damage to the wrist ligament to an extent, affecting fracture healing and wrist function.^2^

Percutaneous internal fixation has been widely used in the clinical treatment of scaphoid fractures. The immobilization time after Kirschner wire internal fixation is long, and there is a risk of Kirschner wire loosening and withdrawal, which may influence the clinical effect. Compared to this, percutaneous minimally invasive screw fixation is the more mainstream surgical treatment at present, which can help to compress the broken end of the fracture, promote fracture healing, and start functional exercise early, and has achieved satisfactory results in the treatment of scaphoid fracture. Percutaneous screw internal fixation under fluoroscopy has become increasingly popular in recent years owing to its minimally invasive and short operation time. However, it cannot detect very well the injury of fracture combined with ligament and triangular fibrochondral complex (TFCC), resulting in poor fracture alignment and repeated puncture fixation during the operation.^9^ Arthroscope‐assisted reduction and percutaneous fixation is not only minimally invasive but also ensures the reduction quality, checks the integrity of various structures of the wrist and diagnosis, and treats the associated ligament and cartilage injuries as early as possible.^10^, ^11^ However, the limited operating space in carpal arthroscopy, long learning curve, and the need for complicated equipment and technical requirements result in a longer operation time than closed reduction and percutaneous hollow nail internal fixation under fluoroscopy, and likely also costs patients more. Furthermore, it is still difficult to widely use in clinical practice.^12^

Thus, the purpose of this study was to discuss the clinical effects of percutaneous screw fixations under fluoroscopy and with arthroscopy assistance for treating Herbert B2‐type acute scaphoid fractures with fracture displacement (step or gap) >1 mm, and to provide a suitable surgical method for the treatment of Herbert B2 type acute scaphoid fractures.

## Materials and Methods

### Inclusion and Exclusion Criteria

The inclusion criteria were: (i) fresh and closed fracture, meeting the diagnostic criteria of scaphoid fracture, and imaging examination showed Herbert B2‐type fracture; (ii) time from injury to operation≤14 days; (iii) fracture displacement >1 mm, with surgical indications; and (iv) availability of complete follow‐up data.

The exclusion criteria were: (i) presence of severe systemic diseases such as diabetes mellitus or cardiopulmonary diseases, contraindicating surgery; (ii) personal or relatives' decision to refuse surgery; (iii) psychological or mental abnormalities; (iv) pathological fracture; and (v) combined with other wrist fractures.

This was a retrospective cohort study. From January 2017 to June 2021, 29 patients (22 male and seven female, age range: 23–56 years) with acute scaphoid fracture met the above criteria and were included in this study. Patients were treated with either closed reduction and percutaneous cannulated screw internal fixation under fluoroscopy or percutaneous cannulated screw internal fixation assisted by arthroscopy. The general data of the two groups before operation are shown in Table 1. There was no statistical significance between the two groups with respect to age, sex, and time from injury to operation (*p* > 0.05). It was approved by the ethics committee of our hospital (2016018) and informed consent was obtained from all the patients.

**TABLE 1:** Comparison of general data between the two groups

Indicators	Fluoroscopy (n = 11)	Arthroscopy (n = 18)	*p*
Sex (male/female)	8/3	14/4	0.758
Age (years)	32 (27, 43)	34 (28.75, 43)	0.740
Injury to operation time (day)	5 (3, 7)	4.5 (2.75, 7.25)	0.877
Dominant hand	8	14	0.815
Follow‐up time (m)	10.91 ± 3.27	10.06 ± 2.31	0.417

### Operative Technique

After brachial plexus block anesthesia, the patients were placed in the supine position, with abduction of the affected limb and pronation of the forearm.

#### Operative Procedure of the Fluoroscopy

**Fig. 1:**
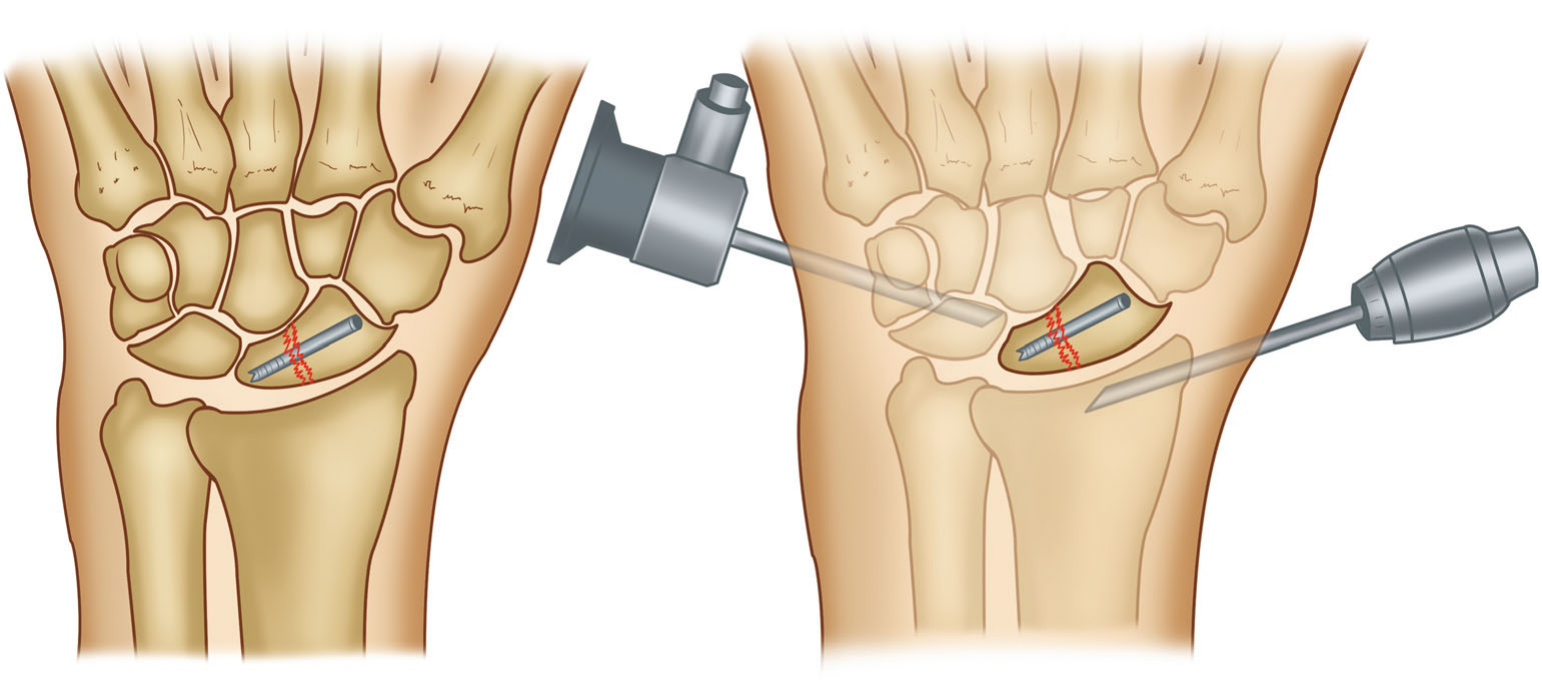
Operative procedure: Closed reduction and percutaneous cannulated screw internal fixation under fluoroscopy; Arthroscopy‐assisted percutaneous cannulated screw internal fixation

In the fluoroscopy group, closed reduction and percutaneous cannulated screw internal fixation under fluoroscopic guidance were carried out (Figure 1A). The needle entry point is at the radial side and distal end of the scaphoid tubercle, that is, the palmar and radial sides of the joint space between the scaphoid and the trapezium bone. Keeping the wrist dorsi flexion and the ulnar deviated, pry and pull the scaphoid through the skin with a Kirschner wire to widen the joint space between the scaphoid and the trapezium bone, to facilitate the insertion of the guide needle. First, traction reduction is carried out, and then the guide needle is inserted. The guide needle with a diameter of 1.0 mm is inserted from the joint surface of the distal scaphoid along the long axis of the scaphoid to the proximal end. Its angle is about 45° to the third metacarpal in the horizontal and sagittal positions. Under the guidance of a C‐arm X‐ray machine, the guide needle must be located in the center of the long axis of the scaphoid. The distal end of the guide needle should reach the bone cortex under the contralateral articular surface. After measuring the length of the guide needle and subtracting about 2 mm, it is the length of the compression screw.

#### Operative Procedure of the Carpal Arthroscope‐Assisted Fixation

In the arthroscopy group, carpal arthroscope‐assisted percutaneous cannulated screw internal fixation was performed (Figure 1B). The surgical procedure was as follows: A pneumatic tourniquet was placed on the upper arm with a frame‐fixed linvatec traction. The elbow was flexed to 90°. Index, middle, and ring finger sleeves were fixed for traction. The traction weight was 5 kg, and the gravity perfusion system was used to inject rinse solution. Mark the 3–4 and 4–5 surgical approaches of the radiocarpal joint, the radial and ulnar midcarpal joint surgical approaches, and the scaphoid joint space approach of the large and small polygonal bone. An arthroscope with a diameter of 27 mm and an inclination angle of 30° (Arthrex, USA) is selected. First, the radiocarpal space is entered through the 3–4 approach, and the intraarticular drainage is performed with a No. 7 guide wire at the 6U approach to check whether the articular cartilage in the radiocarpal joint is intact and whether the TFCC is torn. Enter the arthroscope from the radial wrist joint space, and use a 7‐gauge guide wire for irrigation fluid drainage in the angle‐hook joint space. Use a planer to remove intra‐articular hematoma, and then the probe enters from the ulnar wrist joint space to check scapholunate interval. Use the probe to assist in the reduction of the scaphoid fracture, check the fracture reduction, and adjust the reduction position in time under arthroscopic guidance. Fix one guide wire temporarily after fracture gaps have disappeared. Measure the length of guide wire after ideal fracture restoration under the guidance of the C‐shaped arm X‐ray machine. Drill in the direction of the guide wire with suitable length. Check the fracture reduction and screw position using wrist arthroscopy, and pull out the guide wire after ensuring everything is correct.

### Postoperative Care

After the operation, the short arm plaster support was fixed routinely. Regular activities of the metacarpophalangeal joint and interphalangeal joint were started 2 days after the operation. The suture was removed 2 weeks after the operation, the plaster was removed 2–4 weeks after the operation, and active functional exercise of the wrist joint was started. Radiographic and CT imaging of wrist joint were repeated every 4 weeks until the fracture healed. After the fracture healed, the daily activities and work began to recover.

### Outcome Evaluation

The perioperative data were recorded, and the operation time, intraoperative fluoroscopy times, and fracture healing time of the two groups were compared. During the postoperative 6‐month follow‐up, the modified Mayo wrist function score was used to evaluate the clinical results including pain, function, range of motion, and grip strength. The reduction and healing of bone fracture were evaluated by imaging examination, and any complications were recorded.

### Statistical Methods

SPSS 22.0 software (IBM Corporation Ltd., Armonk, NY, USA) was used for statistical analysis. If the measurement data obeyed the normal distribution, it was expressed as mean ± SD. The independent sample *t*‐test was used for inter‐group comparison. The measurement data that did not conform to normal distribution was expressed as median and interquartile range (IQR), and Wilcoxon rank sum test was used; Fisher's exact probability method was used for countable data. *p* < 0.05 indicated statistical significance.

## Results

### General Results

Both groups of patients successfully completed the operation without vascular or nerve injury. The incision healed well without infection.

### Intraoperative Results

The operation times in the arthroscopy group and fluoroscopy group were 51.50 ± 6.69 min and 56.73 ± 11.48 min, respectively, with no significant difference (*p* > 0.05). Because of tourniquet on upper arm, the operation bleeding volume was about 20 ml in the two groups. The number of fluoroscopies performed in the arthroscopy group was 6.83 ± 1.30, which was less than that in the fluoroscopy group 10.91 ± 2.62 (*p* < 0.05).

### Clinical Results

**Fig. 2:**
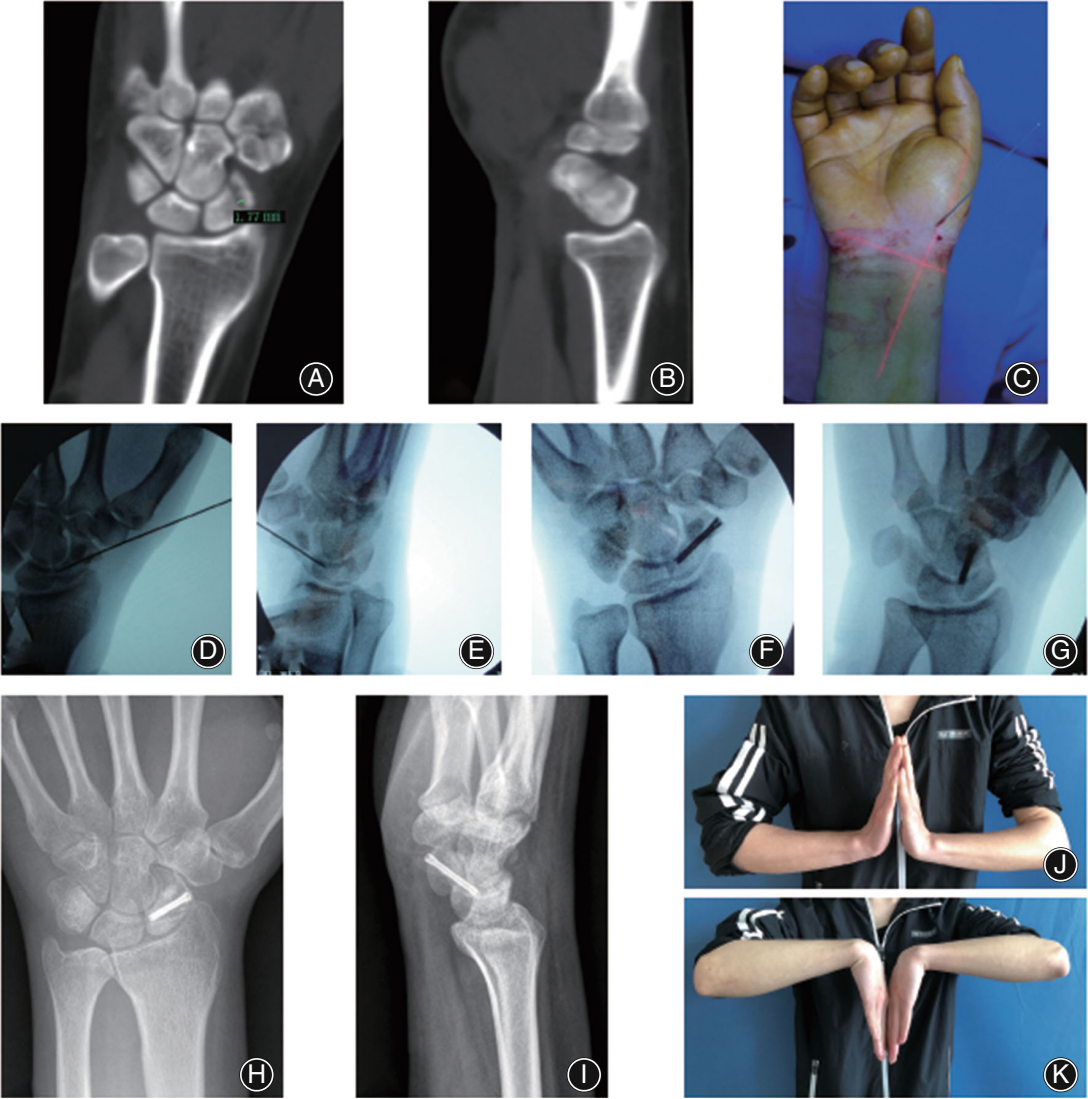
Medical imaging for case 1: A 24‐year‐old male with right wrist pain, swelling, and limited activity for 1 week due to a car accident. (A, B) Preoperative CT showed that the right Herbert B2‐type carpal scaphoid fracture and the separation and displacement of the broken end were 1.77 mm; (C) Closed reduction and temporary guide wire fixation; (D, E) Intraoperative C‐arm fluoroscopy showed that the fracture and guide pin were in good position; (F, G) After screwing in the screw and pulling out the guide pin, C‐arm fluoroscopy showed that the fracture and screw position were good; (H, I) Radiography showed that the fracture line disappeared 12 months after operation; (J, K) 12 months after the operation, the wrist extension and flexion activities were good

**Fig. 3:**
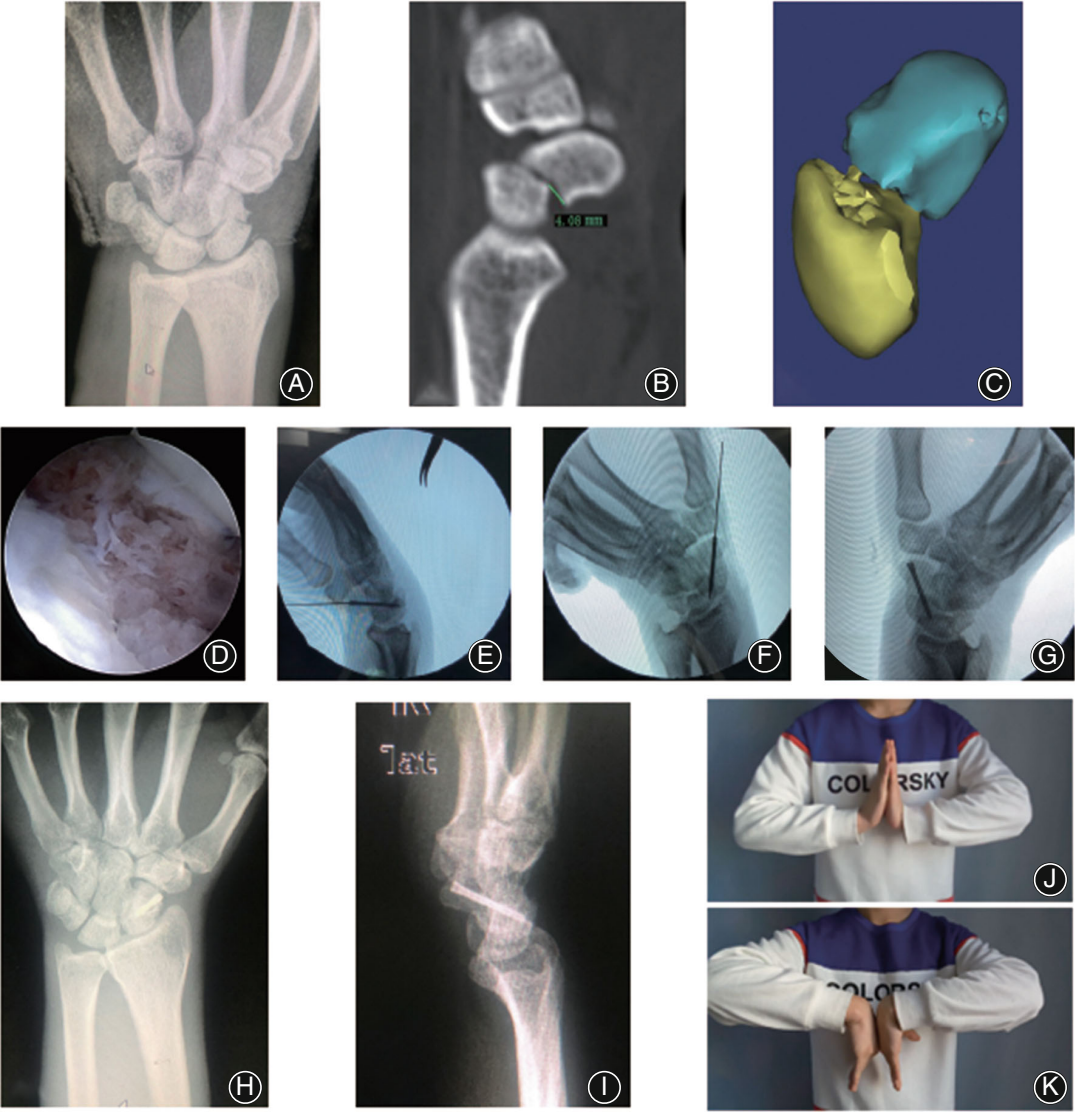
Medical imaging for case 2: A 29‐year‐old male with pain, swelling, and limited activity of the right wrist for 1 day caused by falling from a height. (A) Preoperative CT indicated right carpal scaphoid Herbert B2‐type fracture; (B, C) Preoperative CT and three‐dimensional reconstruction showed that the displacement of carpal scaphoid fracture was obvious, and the fracture step reached 4.08 mm; (D) fracture reduction with arthroscopically assisted procedure; (E, F, G) The screw was inserted along the guide wire, and the fracture and internal fixation were in good position under C‐arm fluoroscopy; (H, I) X‐ray image showed that the fracture line disappeared 15 months after the operation; (J, K) The patient had good wrist extension and flexion at 15 months postoperatively

All fractures in the arthroscopy group healed after the operation, and the fracture healing time was 11.44 ± 1.25 W. Only 10 patients in the fluoroscopy group healed, and the fracture healing time was 13.60 ± 2.32 W. The difference in fracture healing time between both groups was statistically significant (*p* < 0.05). One patient in the fluoroscopy group had nonunion of fracture and healed after bone grafting and internal fixation. No other patient in the study had nonunion of fracture, ischemic necrosis, displacement of internal fixation, or joint degeneration. At the postoperative 6‐month follow‐up, the modified Mayo wrist function score was used to evaluate the clinical results. The wrist function score of patients in the arthroscopy group was 90 (85, 95), while that of patients in the fluoroscopy group was 80 (80, 90) (*p* < 0.05). Typical case representations are shown in Figures 2 and 3.

### Complications

Both groups of patients had no postoperative complications. More detailed statistical data are presented in Table 2.

**TABLE 2:** The results of different surgical methods on wrist scaphoid fracture

Group	Surgery time (min)	Fracture displacement	Number of fluoroscopy (times)	Fracture healing time (w)	Modified Mayo wrist function score
Fluoroscopy	56.73 ± 11.48	2.54 ± 0.77	10.91 ± 2.62	13.60 ± 2.32	80 (80, 90)
Arthroscopy	51.50 ± 6.69	2.86 ± 1.22	6.83 ± 1.30	11.44 ± 1.25	90 (85, 95)
t/Z	1.56	0.890	5.61	3.20	2.74
*p*	0.131	0.092	<0.001	0.003	0.007

## Discussion

### Main Findings of the Study

In this study, scaphoid fractures classified as Herbert B2‐type with displacement of >1 mm within 2W after injury were selected as the surgical indications of carpal arthroscopy‐assisted percutaneous screw internal fixation. We choose the cases of scaphoid fracture with obvious displacement and difficult closed reduction. The number of intraoperative fluoroscopies in the fluoroscopy group was more than the arthroscopy group. The fracture healing time in the fluoroscopy group was longer than that in the arthroscopy group. At the postoperative 6‐month follow‐up, the function scores of Functional Status and ROM in the arthroscopy group were statistically significant than that in the fluoroscopy group. The total operation time of the two groups is similar, and the difference is not statistically significant.

### Treatment Evolution of Scaphoid Fracture

About 80% of the scaphoid surface is covered by articular cartilage, and the dorsal nutrient blood vessels which were derived from the radial artery, entering from the dorsal ridge of the waist to support 70% to 80% of the proximal scaphoid bone.^13^, ^14^ The volar nutrient vessels which originate from the radial artery or the superficial palmar branch of the scaphoid, enter from the scaphoid tubercle and supply 20%–30% of the distal scaphoid. Owing to its anatomical position, the scaphoid waist fracture is the most common among scaphoid fractures. The blood supply of the scaphoid mainly comes from the nourishing vessels of the scaphoid waist, which also explained why it is easy to develop aseptic necrosis and fracture nonunion after scaphoid lumbar fracture. Ahrend *et al*.^15^ studied the distribution of scaphoid volumetric bone mineral density (VBMD), and found that the peripheral region had high VBMD, while the center and waist had the lowest VBMD, which explained why most common fractures appeared in the waist. Apart from this, the small volume of the scaphoid and the abundant ligaments of the peripheral joints have also brought great treatment challenges. Herbert and Fisher^16^ divided scaphoid fractures into stable and unstable fractures, including delayed fracture union and nonunion. Type A is an acute stable fracture, including type A1 which is fractures of the tubercle and type A2 which is an undisplaced “crack” fracture of waist. Type B is an acute unstable fracture, including type B1 with distal oblique fracture, type B2 is displaced or mobile fractures of waist, type B3 is a proximal pole fracture, type B4 is fracture dislocations of carpus, and type B5 is a comminuted fracture. Type C denotes a delayed fracture union, and Type D denotes nonunion. According to this classification, all unstable fractures except type A should be considered for surgical treatment. Displaced fractures of the lumbar and proximal pole of the wrist scaphoid should be treated surgically owing to high incidence of delayed union, nonunion, or ischemic osteonecrosis. Haroon *et al*.^17^ reported that for scaphoid fractures without displacement or with small displacement, plaster fixation has less trauma, reliable curative effect, and relatively low cost. However, the fixation time is long, which may be up to 3 months. Long‐term plaster fixation can lead to complications such as forearm muscle disuse atrophy, wrist stiffness, and osteoporosis. Premature removal of plaster is easy to cause delayed fracture healing or nonunion, causing a variety of complications, which has a significant impact on the functional recovery of the wrist joint. Per literature reports,^18^, ^19^ the overall healing rate of conservative treatment can reach 90%–95%, and the average healing time is 8–12 weeks.^20^, ^21^ It is mainly applicable to distal scaphoid fractures or lumbar fractures with no displacement and displacement less <1 mm. Lumbar fractures and proximal fractures with displacement >1 mm are regarded as contraindications of conservative treatment, because displaced fractures heal slowly and need long‐term fixation. Further, there is a high risk of bone nonunion and traumatic osteoarthritis. Therefore, surgical treatment is often advocated for Herbert B2 acute scaphoid fracture with fracture displacement (step or gap) >1 mm. Open reduction and internal fixation is a common treatment for patients with comminuted scaphoid fracture, old scaphoid fracture, and postoperative nonunion of scaphoid fracture. Although open reduction and internal fixation can achieve a high healing rate, there is the possibility of damaging the palmar ligament during the operation, and there is a risk of further wrist instability. In addition, open reduction may destroy the soft tissue around the scaphoid, prone to scaphoid blood supply disorder, and increase the risk of postoperative scaphoid necrosis, delayed fracture healing, and nonunion.

### New Surgical Techniques of Scaphoid Fracture

In recent years, percutaneous internal fixation for acute scaphoid fracture has been widely used clinically. At present, percutaneous screw internal fixation is often used. The results show that volar percutaneous screw internal fixation can avoid damaging the joint capsule and ligament around the scaphoid and improve the healing rate of fractures.^22^ However, for displaced scaphoid fractures, it is difficult to achieve accurate reduction under percutaneous blind vision. Studies have found that when displaced fractures >1 mm occur, the probability of nonunion is 55%, and the probability of ischemic necrosis is 50%.^23^ The present clinical surgical methods include percutaneous minimally invasive screw fixation, ultrasound‐guided percutaneous screw fixation, wrist arthroscopic percutaneous internal fixation, and robot‐assisted percutaneous internal fixation.^24^ The fracture healing rate of percutaneous minimally invasive Herbert screw fixation under fluoroscopy is more reliable, and the fixation time is shorter, which can help patients recover their function in the early stage. However, this method is also prone to overlooking the possible complications of injury of triangular fibrocartilage complex (TFCC), and the fracture line cannot be directly visualized during the operation. The positioning of a guide needle is difficult and prone to deviation. For fractures with >1 mm displacement, multiple reduction and repeated fluoroscopy are often required, resulting in more radiation injury to doctors and patients. Ultrasound‐guided percutaneous Herbert screw fixation can use ultrasound imaging to display the position and displacement of scaphoid fracture, to optimize the needle entry angle, reduce the number of intraoperative fluoroscopy, and reduce the damage caused by radiation to doctors and patients. However, at the same time, this method also has some limitations: owing to the special tissue structure of the wrist, it often needs multiple ultrasonic explorations to determine the location of the fracture, which has high requirements for doctors' ultrasonic technology. As a minimally invasive surgical technique for scaphoid fracture, percutaneous screw fixation under wrist arthroscopy can directly and accurately observe the scaphoid fracture under the condition of small wound and reduce it. At the same time, it is not easy to damage the blood supply. In addition, it can diagnose and repair other injuries such as TFCC in the wrist joint, which has attracted increasing attention, and is hence considered a better treatment than ultrasound‐guided percutaneous Herbert screw fixation. Whether intra‐articular manipulation will increase the incidence of arthritis is still a controversial issue for wrist scaphoid fractures without displacement. Therefore, in this study, scaphoid fractures classified as Herbert B2‐type with displacement of >1 mm within 2 weeks after injury were selected as the surgical indications of carpal arthroscopy‐assisted percutaneous screw internal fixation. We choose the cases of scaphoid fracture with obvious displacement and difficult closed reduction. In the cases, multiple closed reduction is easy to aggravate injury, destroy the blood supply of scaphoid bone, and increase the operation time and postoperative complications. Robot‐assisted percutaneous internal fixation can increase the success rate of surgery and reduce the incidence of complications. This method also has some limitations such as high initial cost and unsuitability for wider applications. When the patient is fixed on the special positioning fixture, the non‐displaced fracture may be displaced, resulting in secondary injury to the patient. At present, the most commonly used clinical methods are percutaneous minimally invasive screw fixation and percutaneous screw fixation assisted by wrist arthroscopy. It is reported in relevant literature that percutaneous screw internal fixation for scaphoid fracture may have complications such as nerve injury, ischemic osteonecrosis, and bone nonunion.^25^ The follow‐up data of this study showed that the wrist function of all patients recovered well, and there were no related complications. However, the sample size included in this study is small, and there may be some deviation in the results, which needs to be further confirmed by more studies.

Relevant studies have reported that percutaneous screw internal fixation in the treatment of scaphoid fracture can avoid long‐term plaster external fixation, facilitate early postoperative wrist function exercise, and promote the recovery of wrist function.^26^ However, early unprotected activities can lead to fretting at the broken end of the fracture, resulting in delayed fracture healing, nonunion, and other complications. At the same time, early unprotected complete resistance or loaded exercise may also lead to bone nonunion. Therefore, in this study, all cases were fixed using short‐arm plaster support after operation. Active activities of the metacarpophalangeal and interphalangeal joints began 2 days after operation. After 2–4 weeks, the plaster was removed and the active functional exercise of the wrist joint began. The results of this study showed that for patients with fracture displacement >1 mm, the number of intraoperative fluoroscopies in the fluoroscopy group was more than the arthroscopy group. The fracture healing time in the fluoroscopy group was longer than that in the arthroscopy group. At the postoperative 6‐month follow‐up, the function scores of Functional Status and ROM in the arthroscopy group were statistically significant than that in the fluoroscopy group. For patients with fracture displacement >1 mm, percutaneous screw internal fixation under fluoroscopy first requires closed reduction of the fracture, and the effect of fracture reduction needs to be verified through fluoroscopy. At the same time, it needs multiple fluoroscopy during operation to obtain a satisfactory guide pin position, which not only increases the operation time and fluoroscopy exposure of doctors and patients, but also causes bone mass loss. Reducing the firmness of screw fixation and multiple reductions will destroy the blood supply of the navicular bone, aggravate the injury, and prolong the time of fracture healing. In this study, one patient in the fluoroscopy group had fracture nonunion, and the operation and fluoroscopy times were greater than those of other patients in the same group. Fracture nonunion was considered to be related to the above reasons. Wrist arthroscopy‐assisted percutaneous screw internal fixation is more conducive to the improvement of wrist function. Considering that auxiliary wrist arthroscopy can judge whether the fracture fragments overlap, rotate, shift, and fully remove the hematoma and micro fragmented fracture fragments, it can make the fracture separation gap or fracture steps disappear or nearly disappear, to increase the range of motion of early wrist joint and improve wrist function.^27^ However, due to the limited operation space of wrist arthroscopy, the operation time will be increased. The total operation time of the two groups is similar, and the difference is not statistically significant.

### Limitations of the Study

A few limitations of the study include the small number of cases and the short follow‐up period, which could have biased the results to an extent. Therefore, a larger study with longer follow‐up is needed to clarify the safety and effectiveness. The limited operating space in carpal arthroscopy, long learning curve, and the need for complicated equipment and technical requirements result in a longer operation time than closed reduction and percutaneous hollow nail internal fixation under fluoroscopy.

### Conclusion

For Herbert B2‐type acute scaphoid fracture with fracture displacement >1 mm, the arthroscopy‐assisted percutaneous cannulated screw internal fixation has less fluoroscopy times, short fracture healing time, and good recovery effect of wrist function compared to the fluoroscopy. But the operation time of two groups is similar. The arthroscopy‐assisted in the treatment of Herbert B2‐type of acute scaphoid fractures is a good clinical treatment.

## Disclosure

All authors declared no conflict of interest.
